# Anti-inflammatory activity of non-selective PDE inhibitor aminophylline on the lung tissue and respiratory parameters in animal model of ARDS

**DOI:** 10.1186/s12950-023-00337-y

**Published:** 2023-03-16

**Authors:** Petra Kosutova, Pavol Mikolka, Daniela Mokra, Andrea Calkovska

**Affiliations:** 1 Biomedical Center Martin, Jessenius Faculty of Medicine in Martin, Comenius University in Bratislava, Mala Hora 4C, SK-03601 Martin, Slovakia; 2 Department of Physiology, Jessenius Faculty of Medicine in Martin, Comenius University in Bratislava, Mala Hora 4C, SK-03601 Martin, Slovakia

**Keywords:** Non-selective phosphodiesterase inhibitor, Aminophylline, ARDS model, Lung function, Inflammation

## Abstract

Acute respiratory distress syndrome (ARDS) is a common complication of critical illness characterized by lung inflammation, epithelial and endothelial dysfunction, alveolar-capillary leakage, and worsening respiratory failure. The present study aimed to investigate the anti-inflammatory effects of non-selective phosphodiesterase (PDE) inhibitor aminophylline. New Zealand white rabbits were randomly divided into 3 groups: animals with respiratory failure defined as PaO_2_/FiO_2_ ratio (P/F) below < 26.7 kPa, and induced by saline lung lavage (ARDS), animals with ARDS treated with intravenous aminophylline (1 mg/kg; ARDS/AMINO), and healthy ventilated controls (Control). All animals were oxygen ventilated for an additional 4 h and respiratory parameters were recorded regularly. *Post mortem*, the lung tissue was evaluated for oedema formation, markers of inflammation (tumor necrosis factor, TNFα, interleukin (IL)-1β, -6, -8, -10, -13, -18), markers of epithelial damage (receptor for advanced glycation end products, RAGE) and endothelial injury (sphingosine 1-phosphate, S1P), oxidative damage (thiobarbituric acid reactive substances, TBARS, 3-nitrotyrosine, 3NT, total antioxidant capacity, TAC). Aminophylline therapy decreased the levels of pro-inflammatory cytokines, markers of epithelial and endothelial injury, oxidative modifications in lung tissue, reduced lung oedema, and improved lung function parameters compared to untreated ARDS animals. In conclusion, non-selective PDE inhibitor aminophylline showed a significant anti-inflammatory activity suggesting a potential of this drug to be a valuable component of ARDS therapy.

## Introduction

The acute respiratory distress syndrome (ARDS) is a heterogenous syndrome defined by the presence of bilateral radiographic pulmonary opacities, arterial hypoxemia and absence of cardiac failure as a primary cause [1]. It is characterized by alveolar epithelial and lung endothelial damage, which results in increased permeability, pulmonary oedema, and alveolar filling [2]. ARDS develops within one week of a known clinical insult or the onset of new or worsening respiratory symptoms due to a variety of risk factors, including direct (e.g. bacterial or viral pneumonia, gastric aspiration, lung contusion, toxic inhalation, and near drowning) or indirect (e.g. sepsis, pancreatitis, severe trauma, massive blood transfusion, and burn). Despite substantial improvement in understanding the pathophysiology, ARDS remains a common, morbid and life-threatening condition with a mortality of approximately 30% [1, 3–5].

ARDS is a complex condition that involves alveolar and systemic inflammation. Several factors including etiology, host factors (comorbidities and genetics), immunomodulation (e.g. administration of steroids), impact of secondary insults (e.g. ventilator-induced lung injury and nosocomial infection), etc. are likely to affect inflammation in ARDS [6]. In ARDS pathogenesis, the innate immune response plays a critical role. Tissue damage is mediated by many immunological mechanisms that involve neutrophils, macrophages and dendritic cells. Inflammatory insults to the epithelium, alveolar macrophages and vascular endothelium cause an accumulation of protein-rich oedema fluid in the alveoli and consequently hypoxemia due to reduced gas exchange. The participation of alveolar macrophages in the orchestration of inflammation and ARDS clearance is crucial [7]. When alveolar macrophages are activated, neutrophils and circulating macrophages recruit to the lung injury sites. Proteases, reactive oxygen species (ROS), eicosanoids, phospholipids and cytokines are among the bioactive mediators produced by these cells that help to maintain the inflammatory responses.

In ARDS, increased permeability for liquid and protein across the injured lung endothelium results in formation of interstitial oedema. Subsequently, the oedematous fluid rich in proteins, neutrophils and red blood cells translocates to the alveoli, what is typically aided by disruption of the normally tight barrier of the alveolar epithelium [8–10]. Worsening lung compliance causes ventilation-to-perfusion mismatch and right-to-left intrapulmonary shunting which contribute to arterial hypoxemia in ARDS patients. Hand-in-hand with hypoxemia, poor carbon dioxide excretion occurs as a fundamental component of respiratory failure, resulting in increased minute ventilation and increased lung dead space.

Despite intensive research in this field, no pharmacological therapy for ARDS has been shown to reduce evidently the short-term or long-term mortality in ARDS. However, several groups of drugs have a potential to enhance at least partially the clinical status of the patient by reducing the work of breathing (neuromuscular blockers), mitigating inflammation (glucocorticoids), or reducing oxidative stress (antioxidants) [11]. In this context, inhibitors of phosphodiesterases (PDEs) with their wide variety of therapeutic effects may belong to the drugs which could be potentially beneficial in ARDS. Main action of PDEs is based on their ability to split intracellular second messengers cyclic adenosine monophosphate (cAMP) and cyclic guanosine monophosphate (cGMP) to inactive products. The PDE enzymatic superfamily consists of 11 gene families (PDE1 to PDE11), the majority of which contain multiple PDE genes [12]. Thus, some PDE families are cGMP-specific (PDE5, 6, and 9), while others are cAMP-specific (PDE4, 7 and 8), and others hydrolyze both cAMP and cGMP (PDE1, 2, 3, 10, and 11) [13, 14]. Resulting low cAMP concentrations promote inflammation by increasing the production of interleukins (IL)-8, -12, -17, -22, -23, tumor necrosis factor (TNF)α, interferon, and chemokines, while high cAMP concentrations produce an anti-inflammatory response by inducing the synthesis of IL-6 and IL-10 [15]. Effects of PDE inhibitors may be selective, influencing solely one PDE, or non-selective, influencing more PDEs, much as a group of medicaments named methylxanthines. Methylxanthines act through various mechanisms including non-selective PDE inhibition, what is responsible for a wide variety of actions of these drugs [16]. For instance, caffeine is used because of its stimulatory effects on respiration, cognition, and attention while theophylline and theobromine are used in the treatment of bronchial asthma and chronic obstructive pulmonary disease due to their bronchorelaxation, vasorelaxation, and cardiostimulation effects [17, 18]. The complex mechanisms of methylxanthines action are not fully understood. However, they produce bronchodilation and vasodilation by increasing intracellular levels of cAMP and cGMP. Furthermore, methylxanthines suppress the release and action of a variety of pro-inflammatory substances by lowering calcium, acetylcholine, and monoamines in cells [17, 19]. Methylxanthines compete with other purines for receptor binding sites on adenosine receptors because of their similar chemical structures. Thereby, a competitive inhibition of adenosine, an endogenous purine participating in many processes in the airways including bronchoconstriction and chronic inflammation, may cause bronchodilation. Furthermore, methylxanthines promote surfactant production, mucociliary clearance, and reactive oxygen species scavenging [19]. Aminophylline, a methylxanthine and a non-selective PDE inhibitor used in this study, is a combination of theophylline with ethylenediamine in a 2:1 ratio. Although aminophylline is less powerful and has a shorter half-life than theophylline, the addition of ethylenediamine enhances the water solubility and antioxidant effects [20]. Besides pulmonary effects [21, 22], aminophylline demonstrates cardiovascular effects [23, 24], reduces migration of neutrophils, a rich source of elastase, into the lungs, preventing proteolytic pulmonary injury [22], and mitigates protein leakage from the pulmonary capillaries and generation of pulmonary oedema [25], what is likely attributable to an increase in cAMP/cGMP in the lung.

In this study, the anti-inflammatory activity of the non-selective PDE inhibitor aminophylline was evaluated in an experimental model of ARDS prepared in adult rabbits. We hypothesized that intravenous (i.v.) aminophylline may alleviate inflammation and oxidative imbalance and may thus reduce the lung injury and improve the lung function.

## Results

### Lung function parameters

**Fig. 1:**
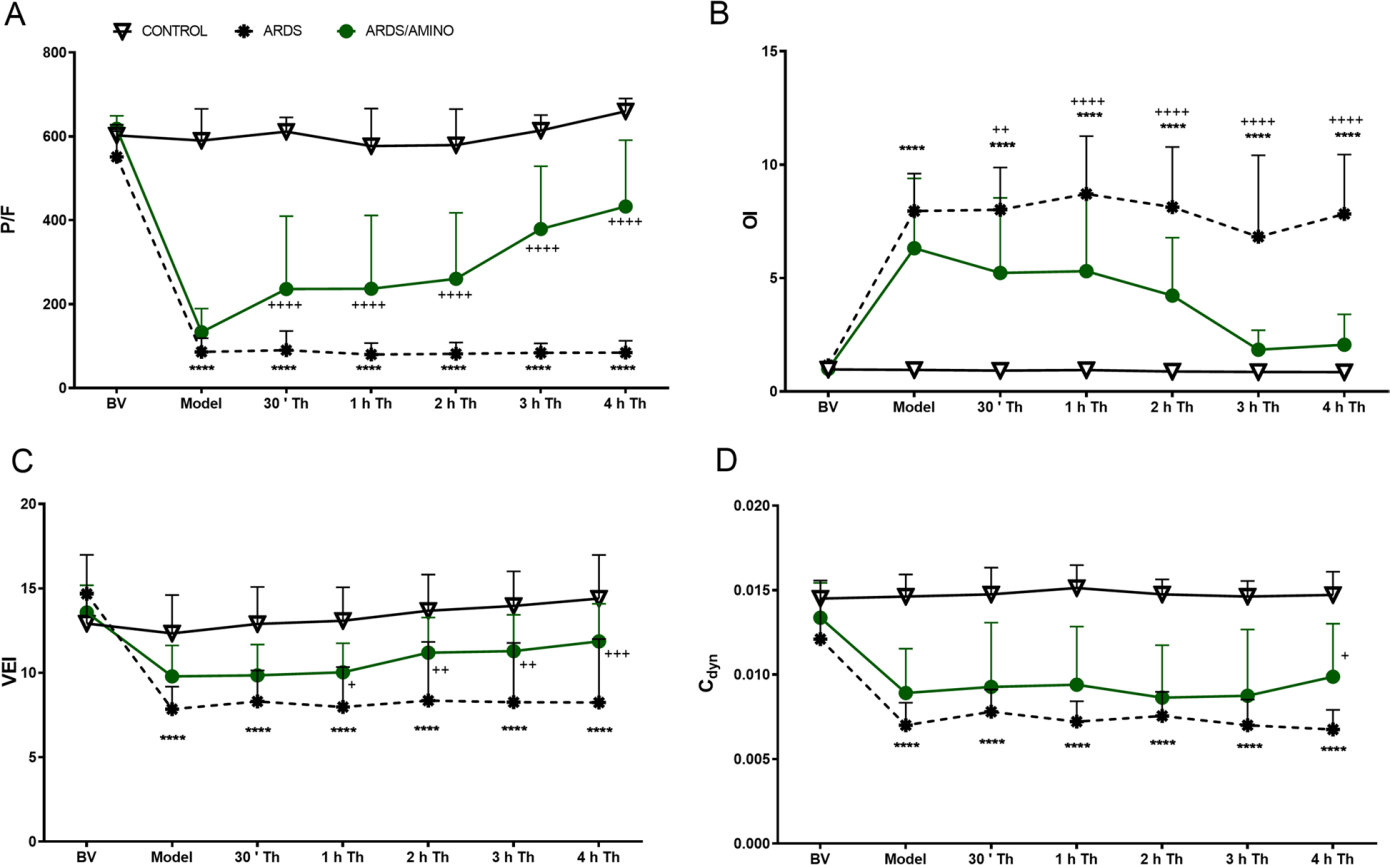
Changes in the respiratory parameters. (**A**) Ratio of the partial pressure of oxygen to the fraction of inspired oxygen (P/F ratio, kPa), (**B**) Oxygenation index (OI), (**C**) Ventilation efficiency index (VEI), and (**D**) Dynamic lung compliance (C_dyn_, mL/kPa) before (basal value, BV) and after induction of ARDS and within 4 h after the therapy administration in the Control group (*n* = 8), ARDS group (*n* = 8), and ARDS group treated with aminophylline (ARDS/AMINO; *n* = 8). Data are presented as mean ± SD. Statistical comparisons: for ARDS *vs.* Control ****p < 0.0001; for ARDS/AMINO *vs.* ARDS ^+^*p* < 0.05, ^++^*p* < 0.01, ^+++^*p* < 0.001; ^++++^*p* < 0.0001

Repetitive lung lavage caused a severe worsening in all observed lung function parameters; the P/F ratio, OI, VEI, C_dyn_, C_stat_, MAP, and Raw had been significantly altered at the time point Model in ARDS group compared to Control (all *p* < 0.001), while P/F ratio < 26.7 kPa was considered for appropriate to reach the moderate ARDS according to the Berlin definition [1]. The deterioration of respiratory parameters in the untreated ARDS group persisted until the end of the experiment (Fig. 1, Table 1). There were no significant differences between the animals in baseline values (BV) of respiratory parameters nor differences in these parameters between the two injured groups (ARDS *vs.* ARDS/AMINO) at the time point of ARDS (all *p* > 0.05).

**Table 1:** Respiratory parameters. Mean airway pressure (MAP, kPa), static lung compliance (C_stat_, mL/kPa) airway resistance (Raw, kPa/L/s), arterial partial carbon dioxide pressure (PaCO_2_, kPa) oxygen saturation (SatO_2_, %) and pH before (basal value, BV), after ARDS induction and within 4 h after the therapy administration in the Control group (*n* = 8), ARDS group (*n* = 8), and ARDS group treated with aminophylline (ARDS/AMINO; *n* = 8). Data are presented as mean ± SD. Statistical comparisons: for ARDS *vs.* Control *p < 0.05, **p < 0.01, ***p < 0.001, **** *p* < 0.0001; for ARDS/AMINO *vs.* ARDS ^+^*p* < 0.05, ^++^*p* < 0.01, ^+++^*p* < 0.001; ^++++^*p* < 0.0001

	**BV**	**Model**	**30' Th**	**1 h Th**	**2 h Th**	**3 h Th**	**4 h Th**
**MAP (kPa)**							
*Control*	0.76 ± 0.06	0.88 ± 0.13	0.86 ± 0.14	0.86 ± 0.16	0.84 ± 0.19	0.85 ± 0.18	0.87 ± 0.19
*ARDS*	0.79 ± 0.08	1.09 ± 0.09 *	1.01 ± 0.13	1.02 ± 0.14	1.06 ± 0.17 *	1.02 ± 0.16	1.06 ± 0.17 *
*ARDS/AMINO*	0.80 ± 0.05	0.90 ± 0.14	0.90 ± 0.12	0.91 ± 0.19	0.94 ± 0.15	0.92 ± 0.14	0.87 ± 0.11 ^+^
**C**_**stat**_** (mL/kPa)**							
*Control*	0.016 ± 0.002	0.016 ± 0.002	0.016 ± 0.002	0.017 ± 0.001	0.017 ± 0.001	0.017 ± 0.001	0.016 ± 0.002
*ARDS*	0.013 ± 0.003	0.008 ± 0.001 ****	0.009 ± 0.002 ****	0.008 ± 0.002 ****	0.009 ± 0.002 ****	0.009 ± 0.002 ****	0.008 ± 0.001 ****
*ARDS/AMINO*	0.016 ± 0.002	0.011 ± 0.002	0.011 ± 0.004	0.012 ± 0.003 ^++^	0.011 ± 0.003	0.011 ± 0.002	0.011 ± 0.003 ^+^
**Raw (kPa/L/s)**							
*Control*	4.51 ± 0.79	4.62 ± 0.91	4.94 ± 1.02	4.44 ± 0.83	4.70 ± 0.77	4.51 ± 0.66	4.61 ± 0.80
*ARDS*	4.29 ± 1.95	12.55 ± 4.03 ****	14.70 ± 5.06 ****	15.22 ± 5.18 ****	14.45 ± 5.37 ****	15.14 ± 5.56 ****	17.38 ± 6.41 ^****^
*ARDS/AMINO*	4.72 ± 0.89	10.17 ± 6.43	8.36 ± 3.84 ^+++^	8.69 ± 4.57 ^+++^	10.18 ± 4.38 ^+^	9.90 ± 4.80 ^++^	9.74 ± 4.97 ^++++^
**PaCO**_**2**_** (kPa)**							
*Control*	4.39 ± 0.67	4.54 ± 0.63	4.34 ± 0.58	4.29 ± 0.56	4.08 ± 0.64	4.04 ± 0.57	4.00 ± 0.70
*ARDS*	3.67 ± 0.86	6.76 ± 1.35 ****	6.56 ± 1.51 ****	6.50 ± 1.34 ****	6.06 ± 1.59 ****	6.19 ± 1.69 ****	6.28 ± 1.92 ****
*ARDS/AMINO*	4.22 ± 0.43	6.07 ± 1.24	6.01 ± 1.31	6.01 ± 1.20	5.55 ± 1.32	5.39 ± 1.31	5.10 ± 1.18 ^+^
**SatO**_**2**_** (%)**							
*Control*	99.90 ± 0.00	99.89 ± 0.03	99.90 ± 0.00	99.89 ± 0.03	99.89 ± 0.03	99.89 ± 0.03	99.89 ± 0.03
*ARDS*	99.89 ± 0.04	95.56 ± 2.43 **	94.13 ± 1.44 **	91.03 ± 13.19 ****	92.11 ± 8.96 ****	94.37 ± 3.57 ****	93.44 ± 4.06 ****
*ARDS/AMINO*	99.90 ± 0.00	97.96 ± 1.72	98.33 ± 1.29 ^+^	98.05 ± 1.72 ^++++^	98.73 ± 1.36 ^++++^	99.24 ± 0.99 ^++^	99.44 ± 0.67 ^++++^
**pH**							
*Control*	7.49 ± 0.07	7.46 ± 0.09	7.41 ± 0.05	7.37 ± 0.03	7.32 ± 0.05	7.27 ± 0.06	7.24 ± 0.05
*ARDS*	7.54 ± 0.06	7.22 ± 0.07 ****	7.21 ± 0.08 ****	7.19 ± 0.09 ****	7.15 ± 0.10 ****	7.10 ± 0.11 ****	7.03 ± 0.11 ****
*ARDS/AMINO*	7.50 ± 0.07	7.27 ± 0.10	7.23 ± 0.08	7.22 ± 0.07	7.19 ± 0.07	7.18 ± 0.06 ^+^	7.16 ± 0.07 ^+++^

Aminophylline obviously improved the lung function parameters (Fig. 1, Table 1). Significant improvements in P/F ratio, OI, SatO_2_, and Raw were observed in ARDS/AMINO group compared to the ARDS group (all *p* < 0.01) immediately after administration of the therapy (30’ Th), while significant differences in VEI, PaCO_2_, pH, C_stat_ and C_dyn_ occurred later. All the mentioned differences persisted until the end of the 4 h observation period.

### Inflammation and oxidation in lung tissue

**Fig. 2:**
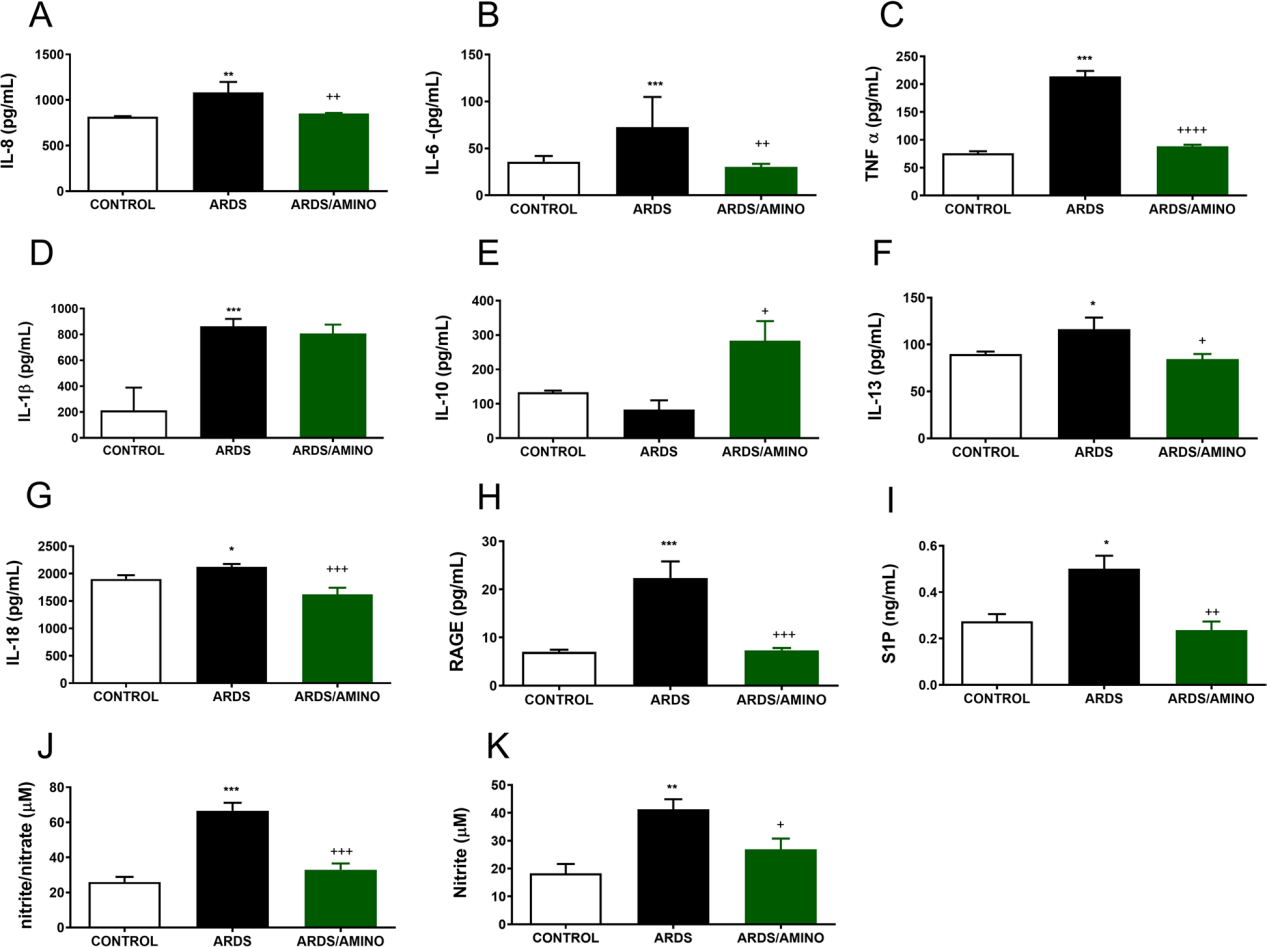
Concentrations of inflammatory cytokines (**A**) IL-8, (**B**) IL-6, (**C**) TNFα, (**D**) IL-11β, (**E**) IL-10, (**F**) IL-13, (**G**) IL-18, (**H**) RAGE (all expressed in pg/mL), (**I**) S1P (expressed in ng/mL), (**J**) nitrite/nitrate and (**K**) nitrite (expressed in NOx micromolar concentration of NOx) in the lung tissue homogenates of healthy ventilated animals (Control; *n* = 8), ARDS animals (*n* = 8) and ARDS animals treated with aminophylline (ARDS/AMINO; *n* = 8) after 4 h of therapy. Data are presented as mean ± SD. Statistical comparisons: for ARDS *vs.* Control *p < 0.05, **p < 0.01, ***p < 0.001; for ARDS/AMINO *vs.* ARDS ^+^*p* < 0.05, ^++^*p* < 0.01, ^+++^*p* < 0.001; ^++++^*p* < 0.0001

Levels of pro-inflammatory cytokines TNFα, IL-1β, -6, -8, -13, -18, RAGE (a marker of lung epithelial injury), S1P (marker of endothelial injury), nitrite/nitrate and nitrite were significantly elevated in the lung tissue in the ARDS group compared to the Control (Fig. 2). Vice-versa, the level of anti-inflammatory cytokine IL-10 was decreased in the ARDS group compared to the Control group. The effect of aminophylline therapy was reflected in decreased levels of the observed markers: for ARDS/AMINO *vs.* ARDS group: IL-8 (*p* = 0.0042), IL-6 (*p* = 0.0013), TNFα (p < 0.0001), IL-1β (*p* = 0.0659), IL-10 (*p* = 0.0140), IL-13 (*p* = 0.0240), IL-18 (*p* = 0.0007), RAGE (*p* = 0.0003), S1P (*p* = 0.0047), nitrite/nitrate (*p* = 0.0003), nitrite (*p* = 0.0363).

**Fig. 3:**
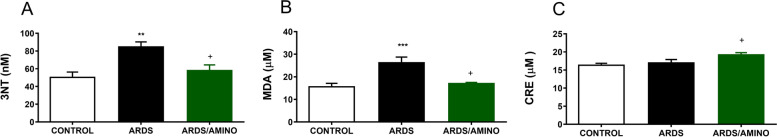
Levels of (**A**) a marker of oxidative modifications of proteins (expressed in nanomolar concentration of 3-nitrotyrosine, 3NT), (**B**) a marker of lipid oxidation (thiobarbituric acid-reactive substances, TBARS, expressed in micromolar concentration of malondialdehyde), and (**C**) total antioxidant capacity (TAC, expressed in micromolar concentration of copper reducing equivalents (CRE)) in the lung tissue of healthy ventilated animals (Control; *n* = 8), in animals with ARDS (ARDS; *n* = 8) and ARDS animals treated with aminophylline (ARDS/AMINO group; *n* = 8) after 4 h therapy. Data are presented as mean ± SD. Statistical comparisons: for ARDS *vs.* Control **p < 0.01, ***p < 0.001; for ARDS/AMINO *vs.* ARDS ^+^*p* < 0.05, ^+++^*p* < 0.001; ^++++^*p* < 0.0001

Both observed markers of oxidative damage, 3-nitrotyrosine (3NT) as an indicator of protein oxidation (*p* = 0.0043), and thiobarbituric acid reactive substances (TBARS) as an indicator of lipid peroxidation (*p* = 0.0008) increased significantly in untreated injured animals compared to controls (ARDS *vs.* Control). Aminophylline therapy decreased the levels of oxidative damage compared to untreated ARDS (3NT, *p* = 0.0150; TBARS, *p* = 0.0112). On the other hand, the total antioxidant capacity (TAC) increased significantly in aminophylline-treated lung compared to untreated ARDS group (*p* = 0.0002) (Fig. 3).

### Lung oedema and protein content in BALF

**Fig. 4:**
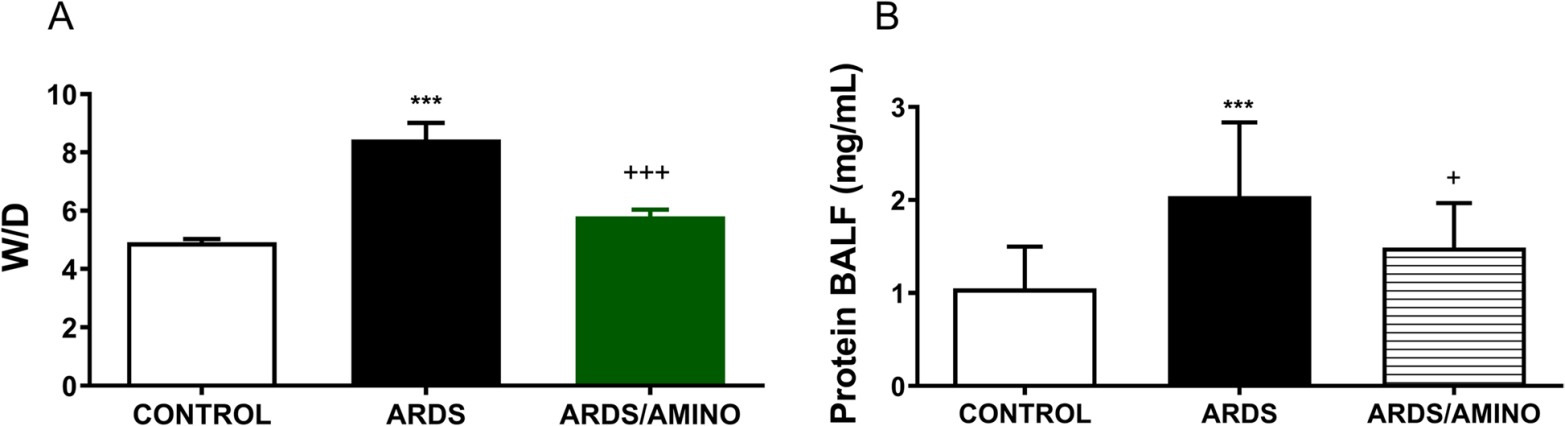
Formation of lung oedema expressed as (**A**) wet-dry lung weight ratio (W/D) and (**B**) protein content in BAL fluid (mg/mL) in healthy ventilated animals (Control; *n* = 8), in animals with ARDS (ARDS; *n* = 8) and in ARDS animals treated with aminophylline (ARDS/AMINO; *n* = 8). Data are presented as mean ± SD. Statistical comparisons: for ARDS *vs.* Control ****p < 0.0001; for ARDS/AMINO *vs.* ARDS ^+^*p* < 0.05, ^+++^*p* < 0.001

Lung oedema expressed as a wet-dry lung weight ratio (W/D) of the lung tissue increased after lavage-induced lung injury compared to controls (*p* < 0.0001), and similarly, total protein content in BALF (*p* < 0.0001) increased for ARDS *vs*. Control. Aminophylline therapy significantly reduced the formation of lung oedema (*p* = 0.0001), as well as the total protein content (*p* = 0.0139) compared to the untreated ARDS group (Fig. 4).

## Discussion

ARDS represents a stereotypic progress through several phases after pulmonary or extrapulmonary insults. At first, alveolar macrophages produce mediators that cause inflammatory cells to accumulate in the lungs and evoke lung tissue damage. Pathologic impairment of vascular permeability in the alveolar epithelial barrier and apoptosis and/or necrosis of type I and II alveolar cells could be the result of induced inflammation and associated release of pro-inflammatory mediators. Pulmonary oedema, surfactant inactivation, and the deposition of dead cells and debris along the alveoli including hyaline membranes are the result of these alterations, which reduce lung compliance and affect gas exchange in the lungs [2, 26]. Inflammatory mediators such as IL-1, IL-6, TNFα, and IL-18 regulate the inflammatory process that is generated by immune cells [27].

PDEs are enzymes catalyzing the metabolism of intracellular cAMP and cGMP that are expressed in a variety of cell types and respiratory diseases [28, 29]. cAMP is therefore increased by inhibiting its degradation by PDEs. PDE inhibitors play a role in airway smooth muscle relaxation and inhibition of cellular inflammation or other immune responses [30] and may also be helpful in treating severe respiratory diseases.

In this study, we focused on the effects of an intravenously administered non-selective PDE inhibitor aminophylline on the inflammatory response, pro-inflammatory cytokine production, oxidative damage and oedema formation, and ultimately on the respiration and gas exchange during the acute phase of experimental ARDS. Ideally, an animal model of ARDS should be a model in which pathologic process maximally mimic clinic situation in patients, such as a neutrophilic alveolitis, deposition of hyaline membranes and formation of microthrombi. In our experiments, rabbits were used as the optimal animal model of ARDS. Larger animals such as pigs and rabbits have IL-8, which is one of the most important neutrophil chemoattractants in humans, and are ideal for complex physiologic measurements [31]. In addition, for evaluating lung function parameters and use of artificial ventilation rabbit lungs are more suitable, the size and diameter of the airways are similar to in the term neonate, than mouse or rat lungs. We are also aware of several interspecies differences in the innate immune response (i.e., in TLR receptors, mononuclear-phagocyte system, NO production, and chemokines and their receptors) that may reduce the translation of results from animal studies to clinics. However, we consider that the selected animal model is appropriately chosen to test our hypothesis.

Lavage-induced imbalance in the alveoli and associated inflammation lead to deterioration of alveolar-capillary membrane integrity and influx of plasma proteins and activated inflammatory cells into the alveoli. This process affects the function of pulmonary surfactant and ventilation-perfusion mismatch, and thus respiration. Repeated lung lavage led to worsening of lung function parameters (P/F, OI, VEI, C_dyn_, MAP, C_stat_, Raw, SatO_2_, PaCO_2_) within minutes after the insult, what is consistent with the findings of other authors [32–34]. In untreated ARDS group, respiratory failure persisted until the end of the experiment probably due to surfactant dysfunction caused by interaction with leaked plasma proteins (albumin and fibrinogen) and/or inflammation [35]. In our study, the therapy with the PDE inhibitor aminophylline improved lung function parameters and gas exchange compared to ARDS animals. We observed a rapid improvement in OI and the P/F ratio within the first 30 min after the aminophylline administration, and this beneficial effect persisted until the end of the experiment. These findings are consistent with previous studies which have shown that nonselective PDE inhibitors (e.g., pentoxifylline, aminophylline) can improve lung function by effective enhancing oxygenation and ventilation parameters [36–39].

In ARDS treatment, it is essential to manage the systemic and also pulmonary inflammatory response. The early phase of ARDS is characterized by neutrophil-mediated inflammation, lung cell injury and apoptosis while neutrophil activation and burst in the lungs play a key role in the progression of ARDS. In our experimental model, increased levels of pro-inflammatory cytokines (IL-8, IL-6, IL-13, IL-18, TNFα, and IL-1β) were observed in the lung tissue of ARDS animals. These results are consistent with previous findings [40, 41]. However, the aminophylline therapy decreased the levels of the observed pro-inflammatory cytokines in the lung tissue compared to the ARDS group. This may be attributable to the fact that increased intracellular secondary messenger cAMP due to activation of adenylyl cyclase affects a broad spectrum of cellular functions; modulates transcription factor nuclear factor-kappa B (NF-κB) and expression of pro-inflammatory cytokines (e.g. IL-1, IL-6, IL-12, IL-13, and TNFα) and regulates expression of anti-inflammatory interleukins [16, 42–44]. In addition, significantly decreased level of anti-inflammatory cytokine IL-10 was found in the lung tissue of ARDS animals, likely due to an imbalance between anti-inflammatory response and serious inflammatory response in ARDS animals [45]. The administration of aminophylline significantly prevented the reduction in IL-10 levels in our study as well as in the study by Elaidy [46]. Elevated IL-10 signaling can inhibit pro-inflammatory cytokine production through direct targeting of immune effector types, but can also indirectly modulate immune function by preventing macrophage and dendritic cell maturation, thus limiting the host's co-stimulatory, antigen presentation, and chemokine secretion capacity of the host [47, 48].

The lung tissue injury may be additionally caused by the oxidation of proteins and lipids due to neutrophil overactivation, especially due to oxidative neutrophil burst. Proteinases, cationic polypeptides, cytokines, and free radicals of reactive oxygen and nitrogen species (RONS) are among the cytotoxic and immune cell activating agents released by neutrophils [49]. After lavage-induced lung injury, significantly increased levels of protein nitrosylation (3-nitrotyrosine, 3NT) and lipid peroxidation products (TBARS) were detected in the lung tissue. Similar oxidation-induced lung damage was confirmed in several studies, demonstrating increased levels of RONS in alveolar spaces during ARDS [50–52]. After aminophylline therapy, the levels of TBARS and 3NT in lung tissue decreased significantly compared to untreated ARDS animals, while, the total antioxidant capacity (TAC) increased significantly after the therapy. The anti-inflammatory and antioxidant effects of nonselective PDE inhibitors at different doses have been demonstrated in various models of injury models [46, 53, 54], since RONS production can be reduced due to achieving a high local concentration of aminophylline in the airways [55].

Other marker evaluated in this study is receptor for advanced glycation end products (RAGE). RAGE is a membrane receptor expressed in alveolar type (AT)-1 epithelial cells of the lung and a marker of epithelial injury [56]. RAGE controls a variety of cellular processes such as cell proliferation and migration, inflammation, apoptosis, and microtubule stabilization [57, 58]. Activation of RAGE plays a role in cell signaling and propagation of the pro-inflammatory response [59–62]. In our study, a significantly higher level of RAGE in ARDS animals was found which was associated with the severity of pulmonary physiological disturbances (P/F ratio and compliance). These results are consistent with the previous study, where RAGE levels correlated with oxygenation [63].

Deterioration of the lung endothelium was demonstrated by sphingosine-1-phosphate (S1P). S1P is highly expressed in the lung endothelium, where it promotes survival and barrier function [64, 65]. S1P’s role as a key regulator of endothelial barrier function is attributed to its signaling through S1P1 & S1P3 that activates downstream Rho GTPases and rearrangement of cytoskeleton [66]. Elevated concentrations of S1P are associated with barrier disruption, as it was observed in ARDS animals in our study. On the other hand, increases in cAMP by inhibition of PDE, e.g. by aminophylline, may improve endothelial barrier functions and support cell–cell junctions [67].

Damage of endothelial and epithelial cells by the above mentioned bioactive compounds, results to increased permeability across the alveolar-capillary membrane and formation of pulmonary oedema. Large numbers of activated neutrophils can damage the alveolar epithelium, probably by the release of toxic intracellular molecules that induce the dissolution of tight junctions [49]. The formation of alveolar oedema containing high molecular weight plasma proteins worsens the gas exchange and increases the risk of disordered repair after extensive alveolar epithelial injury [10, 68, 69]. In our study, the degree of lung oedema was calculated from a ratio of wet and dry lung weight (W/D). The ARDS group had a significantly higher W/D value compared to the control group, indicating increased accumulation of pulmonary fluid in the pulmonary interstitium. Furthermore, the ARDS group had a significantly higher level of total proteins in their BALF. Similar findings had been reported by other authors [33, 68]. In this study, aminophylline therapy decreased the formation of lung oedema and the protein content in BALF compared to the ARDS group. These results are also consistent with the previous studies [70–72].

## Conclusion

In conclusion, aminophylline as a non-selective PDE inhibitor has a potential to be an effective anti-inflammatory drug as it reduced levels of cytokines and oxidative modifications in the lung tissue and prevented the formation of lung oedema. Inhibition of local inflammation alleviated respiratory insufficiency, as indicated by improved lung function parameters. Demonstration of potent anti-inflammatory activity of aminophylline on the lung tissue and enhancing respiration in experimental ARDS as well as long-term positive experience with the clinical use of this drug in the treatment of several respiratory diseases suggest a potential of aminophylline also for ARDS. However, before this treatment can be recommended, further research on optimum dosing, delivery protocol, potential adverse effects etc. should be performed.

## Materials and methods

### Animal instrumentation

Adult New Zealand white rabbits, body weight (b.w.) 2.5 ± 0.2 kg were supplied by the certified animal breeding station (VELAZ, Czech Republic) and handled according to the Federation of European Laboratory Animal Science Associations (FELASA) standards [73]. Animal experimental protocol was approved by the National Veterinary Board of Slovakia and the local Ethics Committee of the Jessenius Faculty of Medicine in Martin, Comenius University.

The animals were instrumented as described previously [74, 75]. After initial sedation, a tracheotomy with an endotracheal cannula insertion was performed and the right femoral artery and vein were cannulated, *a. femoralis* for blood sampling and monitoring of arterial pressure, and *v. femoralis* for continuous intravenous (*i.v.*) anaesthesia infusion (Zoletil, 10 mg/kg/h) and for administration of therapy. The animals were mechanically ventilated (Aura V, Chirana, Slovakia) in a volume-controlled mode with a tidal volume (V_T_) of 6 ml/kg, positive end-expiratory pressure (PEEP) of 5 cm H_2_O, respiratory rate (RR) of 40 breaths per minute (bpm), inspiratory-to-expiratory ratio (I:E) of 1:1 and inspired oxygen fraction (FiO_2_) of 1.0 for the entire duration of the experiment. Respiratory parameters were recorded before (basal value, BV) and after reaching the criteria for Model of lung injury (see below), and 30’, 1 h, 2 h, 3 h and 4 h after administration of therapy. Finally, the animals were sacrificed under deep anesthesia by *i.v.* injection of potassium chloride.

### Experimental protocol

After 15 min of stabilization period (V_T_ 6 ml/kg, PEEP 5 cmH_2_O, RR 40 bpm, I: E 1:1, FiO_2_ 1.0), lung injury was induced by repetitive lung lavages with saline (30 ml/kg, 37° C) through an endotracheal tube in the semi-upright right and left lateral positions of the animal with followed suction. The process was repeated with 2 min intervals of stabilization between the lavages until PaO_2_ in the arterial blood decreased to < 26.7 kPa in two measurements 5 and 15 min after lavage, what equals a moderate degree of ARDS [1].

The animals were randomized to three groups (*n* = 8 for each group): (1) Control group, healthy ventilated animals; (2) ARDS group, animals with ARDS without therapy; (3) ARDS/AMINO group, animals with ARDS treated with single dose of i.v. aminophylline (1 mg/kg b.w. diluted with saline up to volume of 1 ml, Syntophyllin, Hoechst-Biotika, Slovakia), The therapy was administered slowly over 2 min, while Control group and ARDS group received the same volume of saline as placebo. Subsequently, all animals were mechanically ventilated for an additional 4 h with the previously mentioned ventilatory settings.

### Lung function parameters and derived indexes

Electrocardiographic monitoring using subcutaneous electrodes, arterial pressure monitoring through a catheter in *a. femoralis* connected to an electromanometer, tracheal airflow and V_T_ measured by the heated Fleisch head connected to a pneumotachograph were carried out continuously using a multichannel recorder PowerLab 8/30 (AD Instruments, Germany). Partial pressures of oxygen and carbon dioxide (PaO_2_, PaCO_2_), oxygen saturation (SaO_2_) and acid–base balance parameters in arterial blood were measured by a blood gas analyser (RapidLab TM^348^, Bayer Diagnostics, Germany). Ventilation parameters, V_T_, FiO_2_, minute ventilation, RR, peak inspiratory pressure (PIP), PEEP, mean airway pressure (MAP), static and dynamic lung compliance (C_stat_, C_dyn_), and airway resistance (Raw) were measured by in-build sensors and calculated automatically by software of ventilator Aura V (Chirana, Slovakia). The lung function parameters were calculated: P/F as the ratio between arterial PaO_2_ and FiO_2_; oxygenation index (OI) as (MAP x FiO_2_)/PaO_2_; and ventilation efficiency index (VEI) as 3800/[(PIP – PEEP) x RR x PaCO_2_].

### Post-mortem analyzes.

Left lung was lavaged by saline (3 × 10 ml/kg) and the bronchoalveolar lavage fluid (BALF) was centrifuged for 15 min at 1500 rpm. Tissue samples from the right lung were immediately shock frozen and stored at -70° C until biochemical analyzes were performed or used to assess the degree of lung oedema. The levels of inflammatory and oxidation markers were determined in 10% (weight/volume) lung tissue homogenate in 0.1 M phosphate buffer (PBS, pH 7.4). The concentrations of IL-8, IL-6, TNFα, IL-1β, IL-10, IL-13, IL-18, and sphingosine-1-phosphate (S1P) were quantified using the rabbit-specific ELISA kits (USCN Life Science Inc., Wuhan, China), and concentration of receptor for advanced glycation end products (RAGE) was measured by ELISA kit by MyBioSource (San Diego, California, USA), while data were expressed in pg/ml. Oxidative modifications were determined using kits by Cell Biolabs Inc. (USA). OxiSelect™ Nitrotyrosine ELISA Kit for protein oxidation expressed 3-nitrotyrosine in nanomolar concentration (nM 3NT), and OxiSelect™ TBARS Assay Kit for lipid oxidation expressed malondialdehyde in micromolar concentration (μM MDA). Total antioxidant capacity (TAC) was determined using an ELISA kit by Cell Biolabs, Inc. (San Diego, California, USA), while data were expressed in micromolar concentration of copper reducing equivalents (μM CRE). All biochemical analyzes were performed according to the manufacturers' instructions.

The extent of lung oedema was expressed as a wet-to-dry (W/D) lung weight ratio. Strips of the right lung were weighed before and after drying in an oven at 60° C for 48 h to calculate the W/D ratio. The total protein content in BALF was determined by the Bradford colorimetric method, as described previously [76].

### Statistical analysis

Statistical analysis was performed using GraphPad Prism 8.0.1 (USA). Data normality was tested by the Shapiro–Wilk test. All evaluated variables were distributed normally; therefore, one-way ANOVA with Welch’s correction was used to test differences between groups and Tukey's post hoc test to test the parameters with dynamic changes. A *p* value below 0.05 was considered statistically significant. The results are presented as mean ± standard deviation (SD).

## Data Availability

The datasets used and/or analysed during the current study are available from the corresponding author on reasonable request.

## Abbreviations

3NT: 3-Nitrotyrosine
ARDS: Acute respiratory distress syndrome
BALF: Bronchoalveolar lavage fluid
Bpm: Breaths per minute
cAMP: Cyclic adenosine monophosphate
C_DYN_: Dynamic lung compliance
cGMP: Cyclic guanosine monophosphate
CRE: Copper reducing equivalents
C_stat_: Static lung compliance
FiO_2_: Inspired oxygen fraction
i.v.: Intravenous
IL: Interleukin
MAP: Mean airway pressure
MDA: Malondialdehyde
NF-κB: Nuclear factor-kappa B
OI: Oxygenation index
PDE: Phosphodiesterase
PEEP: Positive end-expiratory pressure
PIP: Peak inspiratory pressure
RAGE: Receptor for advanced glycation end products
Raw: Airway resistance
ROS: Reactive oxygen species
RR: Respiratory rate
S1P: Sphingosine-1-phosphate
SaO_2_: Oxygen saturation
TAC: Total antioxidant capacity
TBARS: Thiobarbituric acid reactive substances
TNF: Tumor necrosis factor
VEI: Ventilation efficiency index
V_T_: Tidal volume
